# Molecular Structures of Al/Si and Fe/Si Coprecipitates and the Implication for Selenite Removal

**DOI:** 10.1038/srep24716

**Published:** 2016-04-20

**Authors:** Ya-Ting Chan, Wen-Hui Kuan, Yu-Min Tzou, Tsan-Yao Chen, Yu-Ting Liu, Ming-Kuang Wang, Heng-Yi Teah

**Affiliations:** 1 Department of Soil and Environmental Sciences, National Chung Hsing University, 250 Kuo Kuang Rd., Taichung 40227, Taiwan, R.O.C; 2 Department of Safety, Health and Environmental Engineering, Ming-Chi University of Technology, New Taipei City 24301, Taiwan, R.O.C; 3 Department of Engineering and System Sciences, National Tsing Hua University, Hsin-Chu 30043, Taiwan, R.O.C; 4 Department of Agricultural Chemistry, National Taiwan University, Taipei 10617, Taiwan, R.O.C; 5 Division of Environmental Studies, Graduate School of Frontier Sciences, The University of Tokyo, 332 Building of Environmental Studies, 5-1-5 Kashiwanoha, Kashiwa City, Chiba 277-8563, Japan

## Abstract

Aluminum and iron oxides have been often used in the coagulation processes during water purification due to their unique surface properties toward anions. In the presence of silica, the coprecipitation of Al/Si or Fe/Si might decrease the efficiency of wastewater purification and reuse. In this study, surface properties and molecular structures of Al/Si and Fe/Si coprecipitates were characterized using spectroscopic techniques. Also, the selenite removal efficiency of Al/Si and Fe/Si coprecipitates in relation to their surface and structural properties was investigated. While dissolved silicate increased with increasing pH from Fe/Si coprecipitates, less than 7% of silicate was discernible from Al/Si samples over the range from acidic to alkaline conditions. Our spectroscopic results showed that the associations between Al and Si were relatively stronger than that between Fe and Si in coprecipitates. In Al/Si coprecipitates, core-shell structures were developed with AlO_6_/AlO_4_ domains as the shells and Si frameworks polymerized from the SiO_2_ as the cores. However, Si framework remained relatively unchanged upon coprecipitation with Fe hydroxides in Fe/Si samples. The Si core with Al shell structure of Al/Si coprecipitates shielded the negative charges from SiO_2_ and thereby resulted in a higher adsorption capacity of selenite than Fe/Si coprecipitates.

Silica (SiO_2_) has been widely found in the effluent discharged from the chemical-mechanical-planarization (CMP) process of semiconductor manufacturing, and it showed substantial stability in wastewater since it has a very low point of zero charge, suggesting that it could be the hardest one to remove in conventional wastewater treatment plants by aggregation-sedimentation^1^,^2^. The residual SiO_2_ in wastewater might decrease the efficiency of wastewater reuse as it would clog water pipes, decrease thermal conductivity of boilers, and especially block the reverse osmosis membranes^3^. As a conventional method, SiO_2_ is often removed from wastewater by coagulating/flocculating (i.e., adsorption and/or coprecipitation) with aluminum (Al) or iron (Fe) salts^4^,^5^. Due to the nano-scale particle size and the highly negative surface charges, however, the stability of Al/Si and Fe/Si coagulation is susceptible to change in various polymerization conditions^5^,^6^,^7^,^8^.

The stability of Al/Si and Fe/Si coagulation is in relation to the surface and structural properties of coprecipitates, which would vary with various environmental factors such as temperature, concentration of Si and Fe/Al, pH, and aging time^8^,^9^,^10^,^11^,^12^. While coprecipitated with Fe hydroxides, for example, SiO_2_ would decrease the surface charges of Fe/Si coprecipitates with increasing pH and aging time^13^. However, the surface charges of Al/Si coprecipitates tended to increase with increasing initial concentration of Al hydroxides^12^. The promoted surface charges were derived from Al that hydrolyzed and then adsorbed or precipitated onto SiO_2_^9^. The significant interaction between Al hydroxides and SiO_2_ differentiated the sorption behaviors of Al/Si coprecipitates for nutrients, trace and heavy metals like Cd^2+^ and Ca^2+^ from that of pure Al hydroxides^14^. For Fe/Si coprecipitates, however, the Cd^2+^ removal efficiency was indistinguishable from that of pure Fe hydroxides^14^,^15^. Collectively, the coagulation between Al/Fe with silica might substantially change the surface attributes of coprecipitates and further alter the mechanisms of metal and nutrient retention on Al and Fe hydroxides. However, the mechanistic mechanisms at the molecular scale about how silica affects structural attributes of Al/Si and Fe/Si coprecipitates and the subsequent sorption behaviors have been still unclear.

The interactions of Al(III) or Fe(III) with SiO_2_ on coprecipitate surfaces have been determined qualitatively and quantitatively using surface models, including complexation/precipitation and triple-layer models^13^,^15^,^16^. However, the related mechanic mechanisms, especially at molecular scale, could only be determined using spectroscopic methods. For example, the interaction between Al(III) and SiO_2_ was classified as adsorption, surface-enhanced precipitation, and aluminosilicate precipitation according to the results of nuclear magnetic resonance (NMR) spectroscopy^17^,^18^. The Fourier-transform infrared spectrometer (FT-IR) was employed to determine the elemental binding between Fe and SiO_2_, and the results showed a replacement of Si by Fe on the Fe/Si coprecipitates^19^.

In this study, we aimed to determine the stability Al/Si and Fe/Si coprecipitates in relation to their surface and structural characteristics and further to characterize how such structural changes affect selenite removal. The reason to choose selenite as the target sorbate is that selenite is a common ingredient coming with SiO_4_ in wastewater discharged from the semiconductor manufacturing^20^. In addition, selenium is of environmental interest on account of the limited extent between biologically metabolically required and toxic effect concentrations in many organisms^21^. Wherein, selenite may threaten the environmental ecosystems seriously due to its low toxic doses and bioavailability in water^22^. In this study, we performed several spectroscopic analyses including powder X-ray diffraction (PXRD), transmission electron microscope (TEM), FT-IR, NMR, X-ray absorption spectroscopy (XAS), and X-ray photoelectron spectroscopy (XPS) to determine the molecular structural attributes for the Al/Si and Fe/Si coprecipitates. Subsequently, pH-dependent and isotherm sorption experiments of selenite on Al/Si and Fe/Si coprecipitates were performed to determine the selenite removal efficiency among individual coprecipitates. The combination of the structural characterization and sorption results was used to identify factors that can influence the stability of coprecipitate structures and the magnitude of selenite removal.

## Results

### Silicate Dissolution from Al/Si and Fe/Si coprecipitates

**Figure 1:**
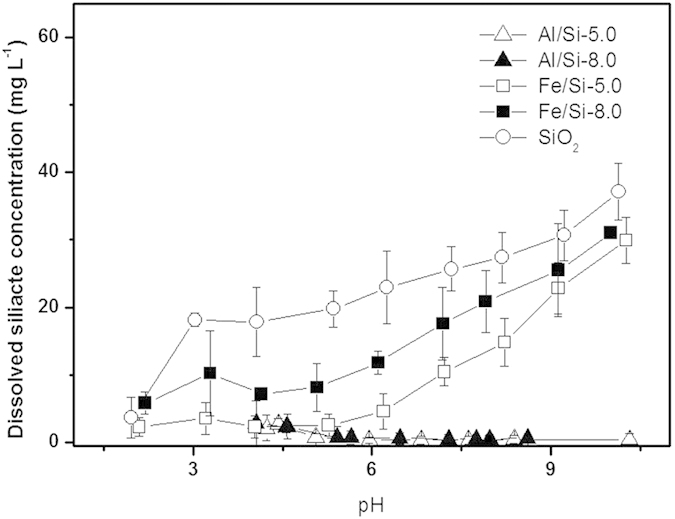
Trends of silicate dissolution from Al/Si and Fe/Si coprecipitates synthesized at pH 5.0 (Al/Si-5.0, Fe/Si-5.0) and 8.0 (Al/Si-8.0, Fe/Si-8.0) under an electrolyte concentration of 0.01 M NaNO_3_.

Trends of silicate dissolution from Al/Si and Fe/Si coprecipitates synthesized at pH 5.0 and 8.0 in 0.01 M NaNO_3_ (Al or Fe/Si-5.0 and -8.0) were shown in Fig. 1. In raw silica solids (SiO_2_), the silicate dissolution tended to increase with increasing pH, agreeing with the previous report^9^. There was a significant amount of dissolved Al or Fe under acidic conditions. However, only less than 0.15% of total added Al in Al/Si samples and 0.08% of total added Fe in Fe/Si samples was found at pH > 5.1 and 3.3, respectively (Figure S1). Such results indicated SiO_2_ was surrounded by AlO_6_ or FeO_6_. While SiO_2_ coprecipitated with Fe hydroxides, the amounts of silicate dissolution was general alleviated among all tested pH, especially for the Fe/Si samples coprecipitated at pH 5.0 (Fe/Si-5.0). Noteworthily, the silicate dissolution was essentially inhibited in Al/Si coprecipitates. For example, while 74%, 60%, and 62% of silicate was dissolved from SiO_2_, Fe/Si-5.0, and Fe/Si-8.0 at pH 10, no discernable silicate dissolution was found for Al/Si samples at alkaline condition. Furthermore, only 5 to 7% of silicate was dissolved from Al/Si-5.0 and Al/Si-8.0 at pH < 4.5.

### Surface and structural properties of Al/Si and Fe/Si coprecipitates

#### BET surface area, PXRD, FE-SEM of Al/Si and Fe/Si coprecipitates

**Figure 2 f2:**
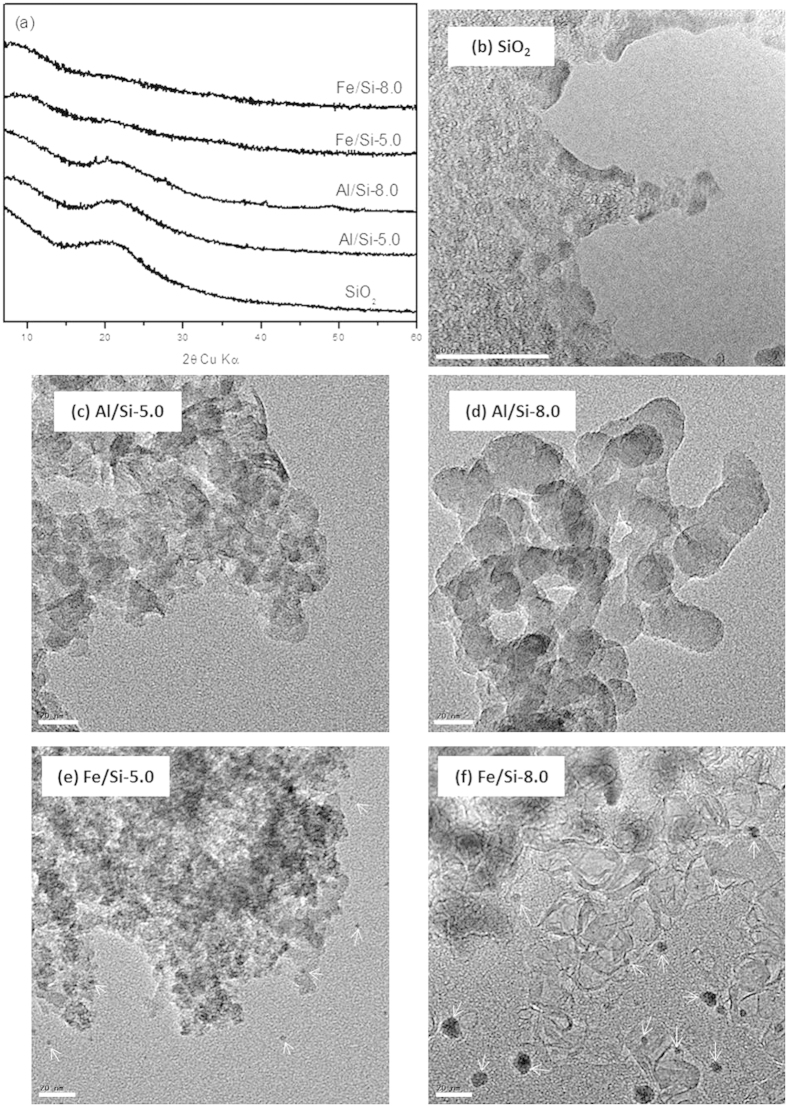
PXRD patterns and TEM images of raw SiO_2_ and Al/Si, Fe/Si coprecipitates synthesized at pH 5.0 and pH 8.0 (Al/Si-5.0, -8.0, Fe/Si-5.0 and -8.0). The white bars represent 20-nm scales and the white arrows pointed out the plausible Fe precipitates.

The BET surface area (S_BET_) for Fe hydroxides (283 and 284 m^2^ g^−1^ for Fe-5.0 and Fe-8.0) are greater than that of Al hydroxides (54 and 158 m^2^ g^−1^ for Al-5.0 and Al-8.0), wherein the Al-5.0 sample showed the lowest S_BET_ (Table S1). While coprecipitated with silica, the S_BET_ for the Al/Si samples were increased substantially. Especially for the Al/Si-5.0 sample, its S_BET_ (155 m^2^ g^−1^) was almost three times greater than that of pure Al hydroxides. In Fe systems, however, the S_BET_ was slightly decreased while coprecipitated with SiO_2_. Figure 2(a) showed the PXRD patterns for Al/Si and Fe/Si samples, which indicated that all samples were non- or poorly crystalline. For Al/Si and SiO_2_ samples, the broad peak centered at 4.1 Å (2θ = 23°) demonstrated the presence of SiO_2_ structures^23^,^24^. However, no discernable peak was found in Fe/Si samples. While no discrete phase was found in Al/Si samples (Fig. 2c[Fig f2]d), there were significant precipitates (dark spots in Fig. 2e,f) in Fe/Si samples. The single phase in Al/Si sample implied a strong association between Al and SiO_2_ surfaces. On the other hand, Fe precipitates and SiO_2_ might form separate phases in Fe/Si systems.

#### Point of zero charge (PZC) of Al/Si and Fe/Si coprecipitates

**Figure 3:**
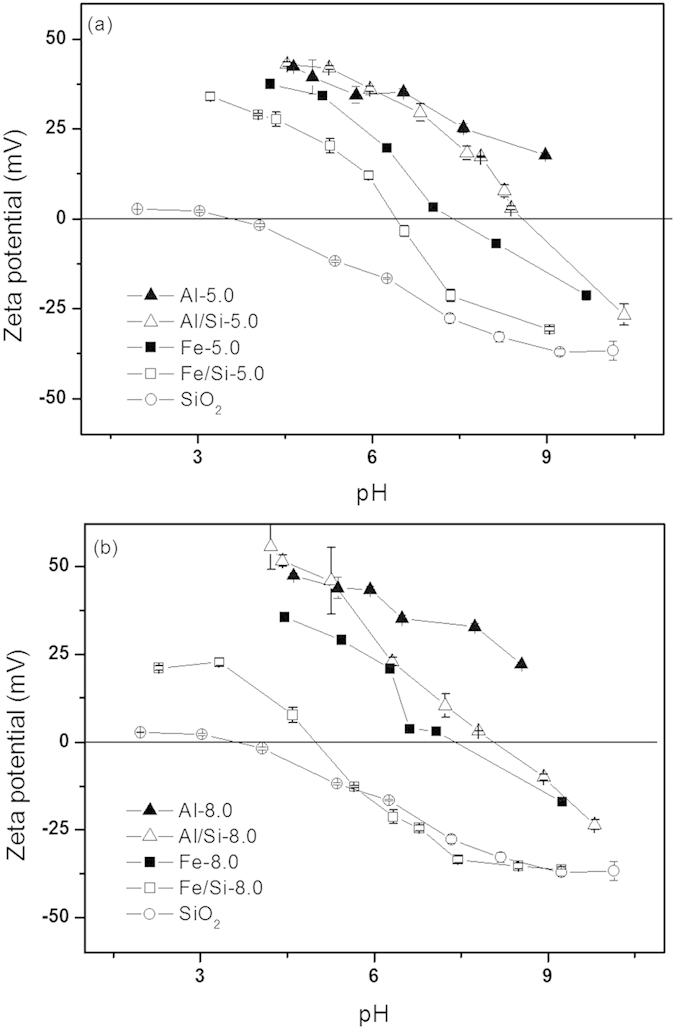
Results of zeta potential for SiO_2_, Al-, Fe-hydroxides, and Al/Si, Fe/Si coprecipitates synthesized at (**a**) pH 5.0 and (**b**) pH 8.0 under an electrolyte concentration of 0.01 M NaNO_3_.

Trends of zeta potential as a function of pH for pure hydroxides and that coprecipitated with silica are showed in Fig. 3. In general, the coprecipitation with silica tended to decrease the PZC for both Al and Fe hydroxides. While hydrolyzed at pH 5.0, for example, the PZC of Fe hydroxide was 7.4, and that was decreased to 6.4 for the Fe/Si-5.0 sample. Similar trend was also found in the systems that hydrolyzed and coprecipitated at pH 8.0 (Fig. 3b). From the perspective of coprecipitation pH, however, the PZC for samples prepared at pH 8.0 were lower than that prepared at pH 5.0. For example, the PZC of Fe/Si-8.0 is 2.4 units less than that of Fe/Si-5.0.

#### FT-IR spectra of Al/Si and Fe/Si coprecipitates

**Figure 4:**
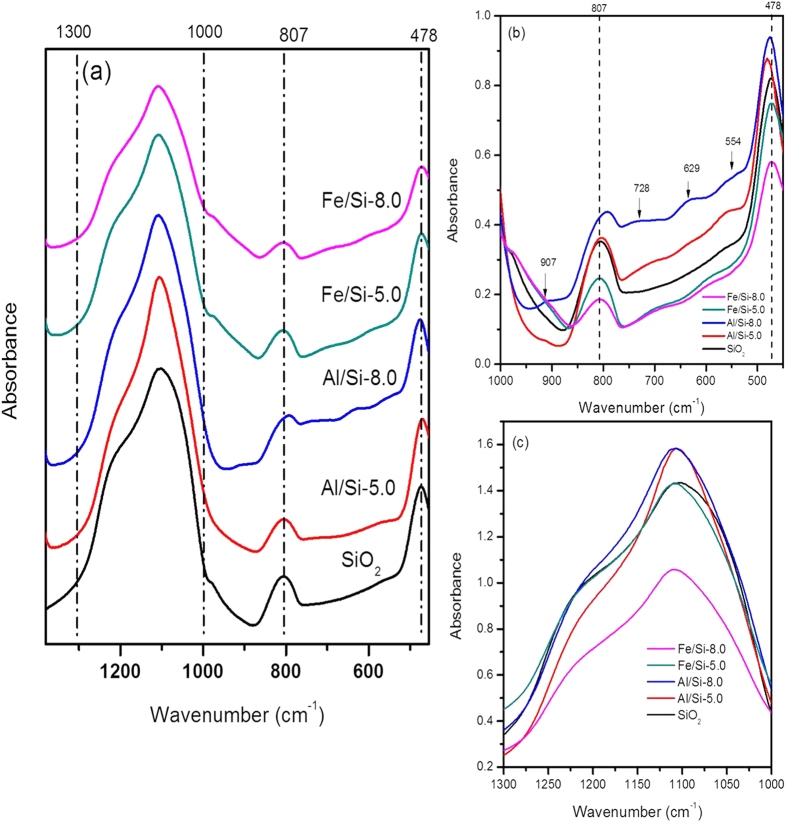
The FT-IR spectra ranged from (a) 450–1380 cm^−1^ and the inlet from (b) 450–1000 cm^−1^ as well as (c) 1000–1300cm^−1^ for SiO_2_ and Al/Si or Fe/Si coprecipitates synthesized at pH 5.0 or 8.0 under an electrolyte concentration of 0.01 M NaNO_3_, respectively.

The FT-IR signals at 1300–1000, 807, and 478 cm^−1^ was found in both pure silica and coprecipitated samples (Fig. 4a), which could be assigned to asymmetric Si–O–Si stretching of isolated SiO_4_ units, symmetric stretching vibration of Si–O–Si network, and bending Si–O–Si/ O–Si–O, respectively^25^,^26^,^27^,^28^,^29^,^30^,^31^.

As shown in Fig. 4b,c without stack increments in the y axis, the intensities at 1300–1000, 807, and 478 cm^−1^ of the Al/Si samples were generally greater than that of Fe/Si samples. Compared with Fe/Si samples, the shoulders at 907, 728, 629, or 554 cm^−1^ of the Al/Si coprecipitates intensified with increasing pH. Noteworthily, the intensity of Si-related signals (1300–1000, 807, and 478 cm^−1^) for Al/Si samples, especially the Al/Si-8.0 one, was relatively greater than that of Fe/Si samples. In Al/Si samples, we also found the emerging shoulders at 907, 728, 629, or 554 cm^−1^ (Fig. 4b). The 907 cm^−1^ mode may derive from the Al-OH deformation vibration^32^. For the ridge at 728 cm^−1^, it may be caused by stretching vibration of Si–O–Al with AlO_6_ coordination^30^ or the adsorbed tetrahedral AlO_4_^33^,^34^,^35^. For the shoulder of 629 and 554 cm^−1^ that was found in Al/Si-5.0 and Al/Si-8.0 respectively, the signals represented the symmetric stretching Si–O–Al^36^ and/or the AlO_6_ coordination^35^,^37^,^38^. Lain on the fact that the intensity of an absorption band is related to the change of the dipole moment of the bond and the amount of the specific bond present, the greater intensity at such Si-related signals for Al/Si samples suggested more or stronger Si–O or Si–O–Si interaction. The changes in the quantity or intensity for such Si–O or Si–O–Si bonding might be resulted from the interaction between Si and Al as suggested by emerging shoulder showed in the Fig. 4b. While Al was demonstrated to be closely associated with silica in the coprecipitates, the interaction between Fe and Si in Fe/Si coprecipitates was not found due to the similar FT-IR spectra to that of SiO_2_ and the absence of the significant Si–O–Fe vibration at 656 cm^−1^ (Fig. 4)^39^.

#### Solid-state ^27^Al and ^29^Si MAS NMR spectra of Al/Si and Fe/Si coprecipitates

**Figure 5:**
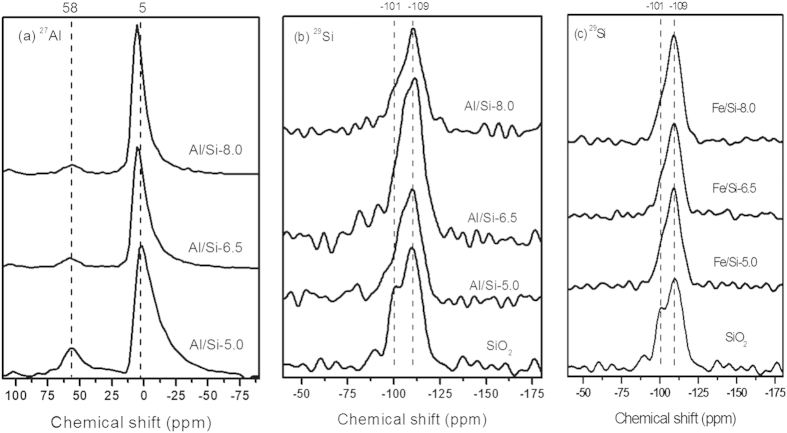
(**a**) 27Al, (**b**) ^29^Si MAS NMR spectra for Al/Si coprecipitates synthesized at pH 5.0, 6.5, and 8.0 (Al/Si-5.0, -6.5, and -8.0), and (**c**) ^29^Si MAS NMR spectra for the Fe/Si coprecipitates synthesized at pH 5.0, 6.5, and 8.0 (Fe/Si-5.0, -6.5, and -8.0) under an electrolyte concentration of 0.01 M NaNO_3_, respectively.

Solid-state ^27^Al and ^29^Si MAS NMR spectra for the Al/Si and Fe/Si coprecipitates were shown in Fig. 5. The ^27^Al NMR spectra for the Al/Si coprecipitates synthesized at various pH (Fig. 5a) showed a discernible crest at 58 ppm and a significant peak around 5 ppm. The feature at around 58 and 5 ppm suggested tetrahedral (AlO_4_) and octahedral (AlO_6_) Al coordination environments, respectively^40^,^41^. Regarding the peak at 58 ppm, the decreasing intensity with increasing pH indicated a fewer amount of AlO_4_ at alkaline condition, agreed with what Houston *et al.* reported^17^, wherein the amount of AlO_4_ tended to decrease and transform to the precipitated AlO_6_ on the surface of silica as pH increased. Such result was in line with the shift of the 5 ppm peak to the higher magnetic field with increasing pH, which suggested more bonding was formed between Al and Si at higher pH^42^.

The ^29^Si MAS NMR spectra of Al/Si samples in Fig. 5b showed a broad peak at −109 ppm and a shoulder at around −101 ppm, which are contributed by the siloxanes with four bridging oxygen (Q_4_) and silanol sites with three bridging oxygen (Q_3_), respectively^42^. Compared with pure silica, the indiscernible signal at −101 ppm for Al/Si samples might indicate the polymerization of silicate, which transformed the silanol sites on silica to siloxane bonds^43^. Besides the −109 ppm peak, there was a should occurred between −101 to −109 ppm in Al/Si coprecipitates, and the position of such should tended to shift to the low-field chemical shift (i.e., more negative shift) with increasing pH. Two possible explanations may account for the signal between −101 to −109 ppm in Al/Si samples: (1) the Q_3_ polymerization to Q_4_ coordination that caused a signal with chemical shift less than −101 ppm; and (2) the bond formation between Si and Al that shifted the Q_4_ signal to the high-field chemical shift, as one Al bonding to Si would increase the ^29^Si NMR signal by 5 ppm^42^. For Fe/Si coprecipitates, the ^29^Si NMR spectra showed a main peak at −109 ppm with a minor signal at −101 ppm regardless of the changes at pH (Fig. 5c), indicating the predominance of Si polymerization^43^. Given that the shoulders at −101 ppm in Fe/Si coprecipitates remained at similar intensity and position at various pH conditions, the absence of Fe/Si bonding in the coprecipitates was suggested^44^.

#### Si K-edge XAS analysis of Al/Si and Fe/Si coprecipitates

**Figure 6:**
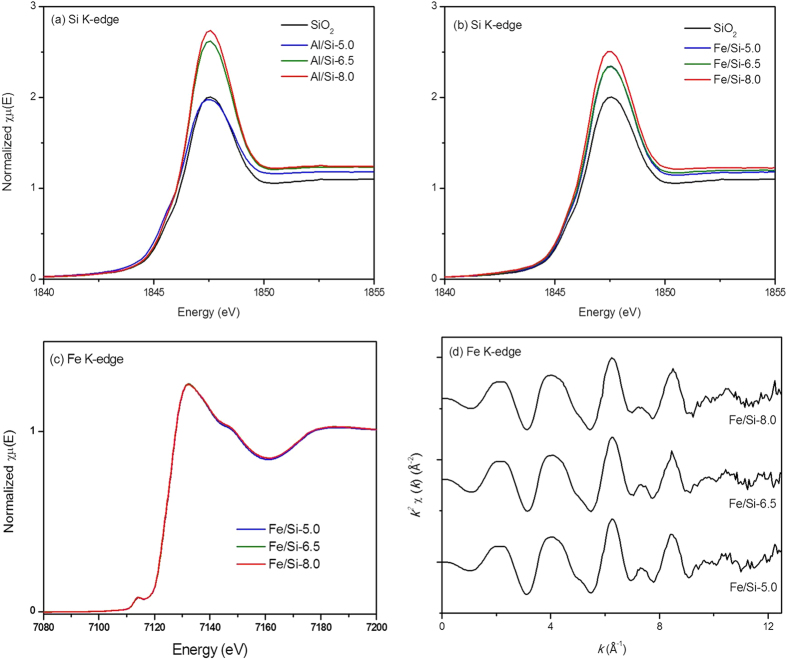
Normalized XANES spectra at Si K-edge for (**a**) SiO_2_, Al/Si coprecipitates (Al/Si-5.0, -6.5, and -8.0), and (**b**) Fe/Si coprecipitates (Fe/Si-5.0, -6.5, and -8.0). The Fe K-edge XANES and *k*^2^-weighted EXAFS data for Fe/Si coprecipitates (Fe/Si-5.0, -6.5, and -8.0) were shown in (**c**,**d**).

Normalized Si and Fe K-edge X-ray absorption near-edge structure (XANES) data for the Al/Si or Fe/Si coprecipitates synthesized at various pH were shown in Fig. 6. For the Si K-edge spectra, all samples showed a sharp white line (WL) centered at 1847.5 eV, wherein the WL intensity for both Al/Si and Fe/Si samples tended to increased with increasing coprecipitation pH (Fig. 6a,b).

While comparing the WL intensity between samples synthesized at same pH, we found a higher WL peak for Al/Si-6.5 than that for Fe/Si-6.5. Similar trend was also observed between Al/Si-8.0 and Fe/Si-8.0 samples, although the WL intensity for the Al/Si-5.0 sample was lower than that of Fe/Si-5.0. Given that the WL peak for Si K-edge XANES is caused by the electron transition from the 1 s to 3p orbitals^45^, whose intensity is related to orbital hybridization^45^ and/or the Si polymerization^46^. Hence, such enhanced WL peaks in Al/Si samples, especially the one coprecipitated at alkaline condition, suggested a higher degree of orbital hybridization between central Si and the surrounding Al and/or the enhanced polymerization of SiO_2_. In the matter of Fe/Si samples, however, we suspected the changes in WL intensity were mainly due to the silica polymerization as no significant difference in the Fe coordination environments was found in the Fe K-edge XANES (Fig. 6c) and extended X-ray absorption fine structure (EXAFS) (Fig. 6d) data for the Fe/Si samples.

#### Element distribution for Al/Si and Fe/Si coprecipitates

The proportions of Al or Fe relative to Si on near surfaces of Al/Si or Fe/Si coprecipitated detected by XPS analysis were tabulated in Table S2. The XPS analysis was used to determine the elemental distribution on near surfaces of coprecipitates rather than the elemental composition for coprecipitated particles. XPS is the most widely used surface analysis technique because it can be applied to a broad range of materials and provides valuable quantitative and chemical state information from the surface of the material being studied^47^. The average depth of analysis for an XPS measurement is approximately 5 nm. On the near surfaces of Al/Si samples, the Al proportions increased from 3.9%–7.2% as pH increased from 5.0–8.0; however, the atomic ratio of Si remained at around 22% regardless of the changes at pH. Such trends resulted in the increasing Al/Si molar ratios from 0.17–0.34 with increasing pH. In contrast with Al/Si samples, the Fe proportion on Fe/Si samples showed insignificant variations as a function of pH, leading to a narrow range in Fe/Si molar ratios from 0.06–0.07. Although the element analysis of XPS was not highly accurate, the present modification in sensitivity factors of XPS rendered it as a reliable quantitative method^48^,^49^,^50^,^51^. The merit of XPS in this study was that it demonstrated the trend that Al tended to precipitate on near surfaces of particles with increasing pH, but the Fe/Si ratios on near surfaces of Fe/Si coprecipitates stayed relatively consistent.

### Selenite removal by Al/Si and Fe/Si coprecipitates

**Figure 7:**
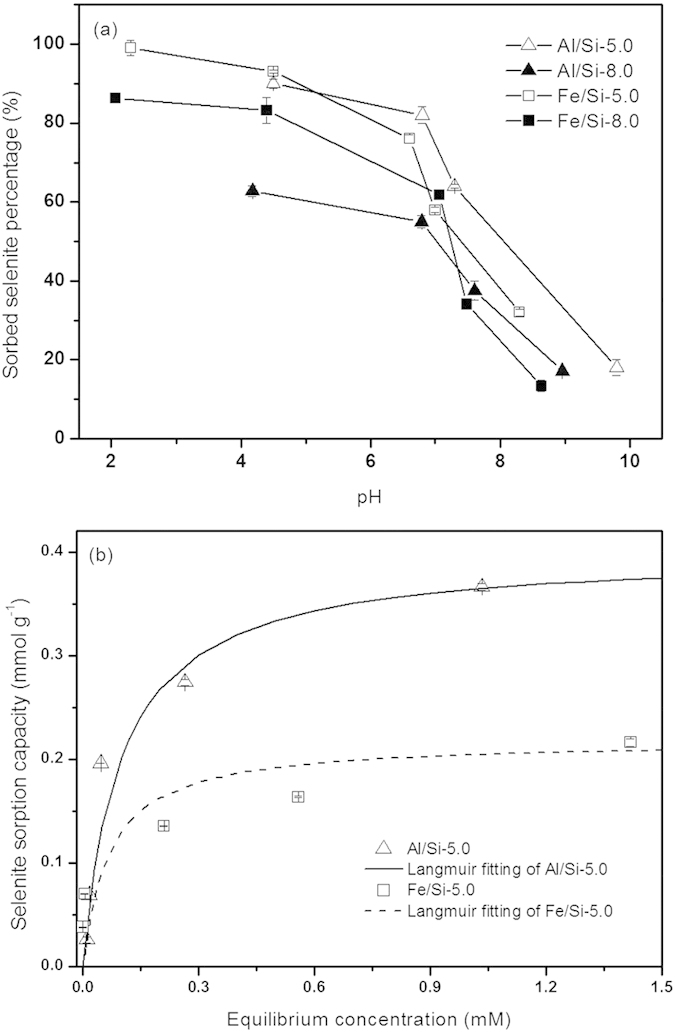
Results of (a) pH-dependent and (b) isotherm sorption of selenite on Al/Si and Fe/Si coprecipitates synthesized at pH 5.0 and 8.0 (Al/Si-5.0, -8.0 and Fe/Si-5.0, -8.0) at 25 °C under a 0.01 M NaNO_3_ background. The Langmuir fitting results showed the maximum adsorption capacities for Al/Si and Fe/Si coprecipitates were 0.40 and 0.22 mmol g^−1^. The coefficients of determination for the fitting are 0.993 and 0.980 (*P* < 0.05, n = 5), respectively.

Given that the pH values for industry and domestic wastewater effluent are generally in the wide range between 1.5–8.5^52^,^53^, it is worthy to examine the pH-dependent removal efficiency of selenite by Al/Si and Fe/Si coprecipitates. As shown in Fig. 7a, for both Al/Si and Fe/Si coprecipitates, the proportion of sorbed selenite decreased as suspension pH was artificially increased. With the exception of Al/Si-8.0 samples, more than 80% of selenite was fixed at pH 4. At pH > 8, however, less than 20% of selenite was fixed on Al/Fe-Si coprecipitates. This observed trend in decreasing sorbed Se with increasing sorption pH could be attributed to the fewer protonated surface sites on coprecipitates that serve as binding sites for selenite^54^,^55^. Given that the Al/Si-5.0 and Fe/Si-5.0 showed relatively greater efficiency for selenite removal than that of Al/Si- and Fe/Si-8.0 among all tasted pH, the samples coprecipitated at pH 5.0 was further used to conduct the isotherm experiments for selenite. As shown in Fig. 7b, the sorption capacity for Al/Si-5.0 (0.40 mmol g^−1^) was 81% higher than that of Fe/Si-5.0 (0.22 mmol g^−1^). As shown in Figure S2, the maximum adsorption capacities of selenite on pure Al(OH)_3_ and Fe(OH)_3_ are 0.41 and 0.28 mmol g^−1^ by Langmuir isothermal model, slightly greater than Al/Si (0.40 mmol g^−1^) and Fe/Si sample (0.22 mmol g^−1^). Given that SiO_2_ particles have almost no sorption capacity for selenite (data not shown), the association of SiO_2_ on Al/Fe hydroxides might decreased the selenite sorption capacity for the Al/Fe-Si coprecipitates. Wherein silica might occupy active sorption sites on Al hydroxides by formed the core-shell structures in Al/Si coprecipitates. In Fe/Si samples, the dissolved silicate might compete for sorption sites on Fe hydroxides with selenite.

## Discussion

Interactions between silica and Al or Fe controlled the surface and structural properties of coprecipitates that further affected the stability of coprecipitates and led to unique selenite sorption results. Compared with Fe/Si coprecipitates, silica in Al/Si coprecipitates seemed to be fixed more rigidly. There was less than 7% of silicate dissolved from Al/Si samples over the range from acidic to alkaline conditions (Fig. 1). That is, coprecipitation with Al hydroxides showed the promise to inhibit the silica reactivity with OH in the solution^16^,^56^. The TEM images (Fig. 2b–f) demonstrated that the single phase in Al/Si sample implied a strong association between Al and SiO_2_ surfaces. On the other hand, Fe precipitates and SiO_2_ might form separate phases in Fe/Si systems. Based on the solubility product constant of 10^−39^ for Fe(OH)_3_ and 10^−34^ for Al(OH)_3_^57^,^58^, Fe hydroxide domains plausibly formed faster than Al hydroxide domains under the same hydrolysis condition. For Al(III) that precipitated slower, the free Al^3+^ could directly bond with Si, resulted in the single phase in Al/Si systems. According to our spectral results (FT-IR, NMR, and XAS in Figs 4,5 and 6), such relatively more stable structure of Al/Si coprecipitates may derive from the bonding formation between Al and Si atoms.

For Al/Si samples, Al^3+^ rapidly hydrolyzed as suspension pH was raised, and the precipitated Al hydroxides may adhere on silica surfaces, wherein the silica served as the active heteronuclei in the coprecipitation processes^59^. While precipitated on silica surfaces, the AlO_6_ domains with a minor amount of AlO_4_ units (Fig. 5a) may interact with the Si framework that polymerized from the SiO_2_ (Figs 4 and 5b), which was evidenced by the stretching vibration of Si–O–Al in FT-IR spectra of Al/Si samples (Fig. 4). Given that the indiscernible interaction between Fe and Si was evidenced by spectral analyses, we proposed Fe hydroxides might form discrete phases with SiO_2_ during coprecipitation. If any bonding between Fe and Si formed, a tetrahedral Fe coordination should be found, which would show the fingerprints of 7131.4, 7137.4, and 7148.2 eV at Fe K-edge XANES spectrum^60^. For our Fe/Si samples, however, the Fe K-edge XANES and EXAFS data (Fig. 6c,d) suggested that the Fe structures were more like poorly crystalline Fe hydroxides^61^. The possible explanation for the discrete Fe phases in our Fe/Si samples is that the coprecipitation process was not performed under thermal condition. Previous studies^44^,^60^ suggested that the Fe-Si interaction might occur when coprecipitates are subjected to thermal treatments.

**Figure 8:**
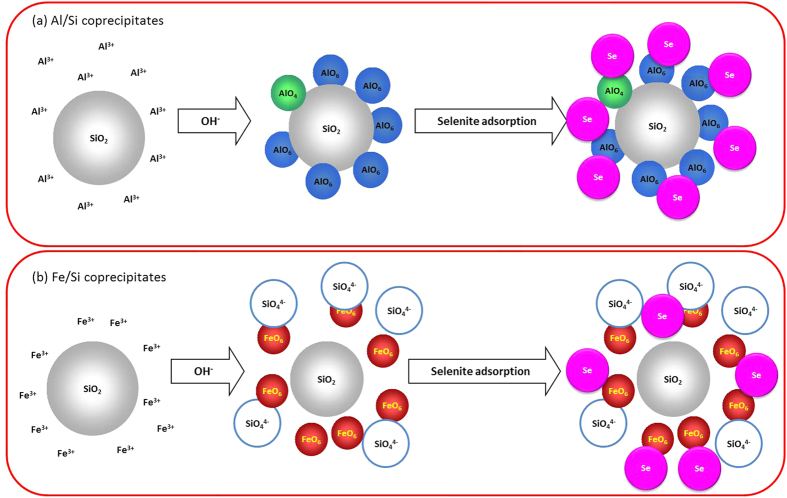
Conceptualized structural development and removal mechanisms of selenite for (a) Al/Si and (b) Fe/Si coprecipitates.

Taken together, our findings allow us to conceptualize the structural development for Al/Si and Fe/Si coprecipitates in Fig. 6. Here, AlO_6_ with minor amounts of AlO_4_ that hydrolyzed from Al^3+^ adhered onto SiO_2_ surfaces by forming the direct bonding of Si–O–Al (Fig. 8a). Such robust structural development suppressed the silicate dissolution. On the contrary, the octahedral Fe domains that grew from Fe^3+^ hydrolysis were loosely associated with SiO_2_ colloids. The relatively isolated SiO_2_ in Fe/Si samples, therefore, led to the more pronounced silicate release (Fig. 8b). The forgoing mechanisms of structural developments were also supported by our XPS data. The Al/Si ratios on the near surfaces of Al/Si coprecipitates were significantly greater than the Fe/Si ratios for Fe/Si samples. The enrichment of Al relative to Si implied that the SiO_2_ might serve as a core with Al attached at SiO_2_ surfaces. This was in line with the core-shell structure that plausible formed during the heterogeneous nucleation of Al hydroxide with SiO_2_ serving as a template^59^,^62^. For Fe/Si samples, however, the trifle amount of Fe relative to Si on the near surfaces of coprecipitates implied the relatively independent phases formed between SiO_2_ and Fe hydroxides.

The trend in decreasing sorbed selenite with increasing sorption pH could be attributed to the fewer protonated surface sites on coprecipitates that serve as binding sites for selenite (Fig. 7a)^54^,^55^. In relation to the coprecipitation pH condition, both Al/Si and Fe/Si samples prepared at pH 5.0 showed the greater removal efficiency for selenite over the tested pH range (Fig. 7a). Such results might be ascribed to the higher PZC values for samples prepared at pH 5.0 than that prepared at pH 8.0 (Fig. 3a). In terms of coprecipitated elements, the Al/Si-5.0 sample showed a relatively superior sorption capacity of selenite than Fe/Si-5.0 (Fig. 7b), which also agreed with the much higher PZC at Al/Si-5.0 than at Fe/Si-5.0 (Fig. 3a). Due to the core-shell structures, the negative charges on silica might be sterically shielded by Al hydroxides. Such steric effect that decreased the exposure of negative charges of silica was rarely achieved in the independent phases between SiO_2_ and Fe hydroxides (Fig. 8). Moreover, while silica occupied the active sorption sites on Al hydroxides by formed the core-shell structures in Al/Si coprecipitates, the dissolved silicate competed for active sites on Fe hydroxides with selenite (Fig. 8)^63^. On the basis of the differences between Al/Si and Fe/Si samples, surface properties that plausibly deduced from structural attributes for coprecipitates showed the promise to influence the extent of selenite removal. Although the association between Al and Si in Al/Si-8.0 was stronger than that in Al/Si-5.0, the surface charges of Al/Si-8.0 derived from OH groups, resulting in less selenite removal than Al/Si-5.0 (Fig. 7a).

Our spectroscopic results for Al/Si and Fe/Si coprecipitates indicated that Al tended to interact with silica and precipitate as AlO_6_ and/or AlO_4_ polyhedra on surfaces of silica particles. Such structural development was more pronounced with increasing coprecipitation pH. However, no discernible interaction between Fe and Si was found in Fe/Si coprecipitates. Silica and Fe hydroxides seemed to occur as independent phases. While the formation of Si–O–Al bonds on Al/Si coprecipitates modified their surface properties from the pure SiO_2_, the independent phases in Fe/Si coprecipitates reserved the original SiO_2_ properties, including silicate dissolution, surface charges, and structures. The selenite removal by Al/Si and Fe/Si coprecipitates could generally be estimated using their PZC values, however the differences in PZC between Al/Si and Fe/Si samples were a consequence of the various structural development. The Al/Si samples showed a relatively rigid coprecipitate structures and a relatively higher selenite sorption capacity than Fe/Si. Thus, we would suggest using Al rather than Fe to remove Si and selenite from the wastewater treatment discharged from semiconductor manufacturing.

## Methods

### Preparation and stability of Al/Si and Fe/Si coprecipitates

Briefly, Al/Si and Fe/Si coprecipitates were synthesized by mixing Al(III) or Fe(III) solutions with suspensions of noncrystalline SiO_2_ (Cab-O-Sil^®^M5 from Cabot Corp., Tuscolca, IL) under a 0.01 M NaNO_3_ electrolyte. Prior to coprecipitation, SiO_2_ suspension in a concentration of 10 g L^−1^ 0.01 M NaNO_3_ was aged at 25 °C under an N_2_ atmosphere for 24 h. Subsequently, an appropriate amount of 0.5 M Al(NO_3_)_3_ · 9 H_2_O or Fe(NO_3_)_3_ · 9 H_2_O was added into the SiO_2_ suspension to achieve a final concentration of 5 mM Al or Fe in 1 g L^−1^ SiO_2_. The mixture was agitated at 300 rpm and pH 5.0 using a pH-stat (TIM865 Titration Manager, Radiometer Analytical) with 0.01 M NaOH or HNO_3_. After 24 h, the suspension was centrifuged (Hitachi, 18 PR-52) at 21,400 × g for 15 min. The collected precipitates were washed three times using double deionized water (DDW > 18 M-cm) to remove excessive salts and then freeze-dried (Millitorr Elemech FD-101) for further analyses. Identical coprecipitation procedures, but with different coprecipitation pH at 6.5 and 8.0, was also conducted. Al/Si and Fe/Si coprecipitates synthesized at pH 5.0, 6.5, and 8.0 were hereafter entitled as Al/Si- or Fe/Si-5.0, 6.5, 8.0. Samples of pure Al- and Fe-hydroxides were also hydrolyzed using identical procedures at pH 5.0 and 8.0 (Al- or Fe-5.0, 8.0), but in the absence of SiO_2_.

The degree of stability of Al/Si or Fe/Si coprecipitates was determined based on the silicate dissolution from the solids. The pH-dependent leaching experiment of silicate from Al/Si or Fe/Si coprecipitates was performed in a suspension at a concentration of 3 g L^−1^ DDW. The suspensions were continuously mixed end-over-end for 24 h at room temperature (25−27 °C) across the pH range from 2 to 10 (adjusted using 1 M NaOH or HNO_3_). The pH was measured after incubation and prior to the centrifugation. Separation of supernatant from the residual coprecipitates was conducted using the centrifugation at 21,400 × g for 15 min. Amounts of silicate in aqueous samples were analyzed using inductively coupled plasma atomic emission (ICP-AES) spectroscopy (Perkin Elmer, Optima 2000DV).

### Surface analyses of Al/Si and Fe/Si coprecipitates

Freeze-dried subsamples of Al/Si and Fe/Si coprecipitates were used to determine the single-point Brunauer-Emmett-Teller (BET) surface areas with a Micromeritics Tristar 3000 gas adsorption analyzer. To understand the morphology of Al/Si and Fe/Si coprecipitates, a drop of the suspension was spread on a thin cellulose film supported by a copper grid. The specimen was dried for a few minutes at room temperature. The TEM images were investigated with a Jeol JEM-2100 ultrahigh resolution transmission electron microscope (accelerating voltage: 200 KV, stability: 2 × 10^−6^ min^−1^). Suspensions of individual coprecipitates in the concentration of 3 g L^−1^ 0.01 M NaNO_3_ were prepared, shaken for 24 h, and used for zeta potentials analysis with Malvern Zetasizer 3000 HS.

To determine elemental compositions on near surfaces of Al/Si and Fe/Si coprecipitates, XPS analyses were performed using a ULVAC-PHI (PHI 5000 VersaProbe/Scanning ESCA Microprobe) with monochromatic Al Kα radiation (hν = 1486.6 eV) for plane-view samples at 45° angle of emission. The X-ray spot size was approximately 100 μm × 100 μm. The pressure in the analytical chamber during spectral acquisition was less than 6.7 × 10^−8^ Pa. The analyzer pass energy was 187.85 eV. The energy step size for high resolution scans was 0.1 eV. Surface concentrations of elements in atomic percent were obtained from spectral deconvolution and fitting of O 1 s, Si 2p, Fe 2p3/2, and Al 2p signals.

### Structural analyses of Al/Si and Fe/Si coprecipitates

We further used PXRD, FT-IR, ^27^Al and ^29^Si solid-state magic angle spinning nuclear magnetic resonance spectroscopy (MAS NMR), and XAS to characterize the structural properties of Al/Si and Fe/Si coprecipitates.

For structural analysis, PXRD patterns were obtained using a diffractometer (PHILIPS X’PERT Pro MPD) with monochromatized and Ni-filtered Cu Kα radiation (λ = 1.5405 Å). The diffractometer was operated at 45 kV and 40 mA.

For FT-IR analysis, spectra were collected using a Bomem, DA8.3 spectrometer with an optical resolution of 2 cm^−1^. Powers for FT-IR pellets were prepared by mixing oven-dried (110 °C) coprecipitates with KBr at a concentration of 5 mg g^−1^. Spectra were acquired in the range from 4000–450 cm^−1^.

The ^27^Al and ^29^Si MAS NMR analysis of coprecipitates was performed in a Bruker DSX-400 NMR solid state high resolution spectrometer, which was operated at 9.4 T with spinning rate varied between 6.4 kHz (7 mm rotor) and 10 kHz (4 mm rotor). The ^29^Si spectra were calibrated using Si(CH_3_)_4_ and acquired at 79.4 MHz with 2.5 μs pluses (45° pulse width) and 7 s recycling times. The ^27^Al spectra were calibrated using AlCl_3(aq)_ and collected at 104.1 MHz with 1 μs pluses (45° pulse width) and 0.5 s recycling times.

The local coordination of Si and/or Fe in Al/Si and Fe/Si samples was characterized using XAS, including X-ray absorption near-edge structure (XANES) and extended X-ray absorption fine structure (EXAFS) spectroscopy. The XAS spectra at Si and/or Fe K-edge were collected at National Synchrotron Radiation Research Center (NSRRC), Hsin-Chu, Taiwan, where the storage ring was operated at 1.5 GeV with a fixed current of 250 mA. An aliquot of freeze-dried sample was mounted in an acrylic sample holder at a thickness calculated to yield unit edge step across the Si and/or Fe K-edge near 1839 and 7112 eV, respectively^64^.

For Si XAS, the wet paste samples were mounted on the holder without cover. The spectra were collected at beamline BL-16A1. The synchrotron radiation that passes through focusing mirrors was detuned by 50% at 50 eV above the Si K-edge. The InSb(111) monochromator was calibrated to 1839 eV based on the first inflection point in the first derivative spectra of elemental Si. Spectra were collected in fluorescence mode with a He_(g)_-filled Lytle detector between −70 to +610 eV relative to the Si K-edge at 1839 eV, using a step size of 0.2 eV across the absorption edge region.

For Fe XAS, the samples were sealed with Kapton tape, and spectra were collected at Wiggler 20 beamline BL-17C1. The Si(111) monochromator energy of a Fe foil was calibrated to 7112 eV monitored during data collection. Samples were analyzed in fluorescence mode using an Ar_(g)_-filled Lytle detector between −200 to +800 eV relative to 7112 eV, using a step size of 0.2 eV in the near edge region (−30–50 eV) and a step size of k = 0.06 Å^−1^ at higher energies^61^.

Multiple XAS scans on each sample were aligned, merged, and processed using the Athena program, an interface to IFEFFIT (version 1.2.10)^65^,^66^,^67^. Self-absorption effects, if they existed, were corrected in the Athena program. Spectra were baseline corrected using a linear pre-edge function between −75 to −10 eV for Si-XAS and −150 to −30 eV for Fe-XAS. The spectral normalization was conducted using a linear or quadratic function between 20–600 eV for Si-XAS and 50–750 eV for Fe-XAS, including a flattening function in the post-edge region^64^. For Fe K-edge EXAFS spectra, backgrounds were removed using a cubic spline fit with nodes defined by the AUTOBKG function in IFEFFIT, and the EXAFS data were extracted from the normalized XAS spectra and converted to their (*k*) function as the *k*^2^-weighted data^61^

### pH-dependent and isotherm sorption of selenite on Al/Si and Fe/Si coprecipitates

The pH-dependent sorption experiments were conducted by mixing 1 mM of selenite reacted with 50 mL the Al/Si-5.0, 8.0 or Fe/Si-5.0, 8.0 coprecipitates at a solid concentration of 3 g L^−1^. The incubations were carried out at 25 °C for 24 h in a 0.01 M NaNO_3_ background as a function of pH from 2.0–10.0. Besides, isotherm sorption experiments of selenite (2 to 0.1 mM) were conducted at pH 5.0 with same other incubation conditions as pH-dependent experiments. After the 24-h incubation, suspensions for both pH-dependent and isotherm experiments were passed through a 0.22 μm pore-size membrane filter (Millipore filter). Selenite in filtrates was analyzed using ICP-AES, and the isothermal results were fitted by Langmuir isotherm model. In the end of sorption experiments, the suspension samples were collected and analyzed using the Se-XAS. The Se-XANES spectra (data not shown) indicated that no oxidation or reduction of selenite was found during the experiments. The differences between the initial and final selenite concentrations were attributed to sorption onto the coprecipitates.

## Additional Information

**How to cite this article**: Chan, Y.-T. *et al.* Molecular Structures of Al/Si and Fe/Si Coprecipitates and the Implication for Selenite Removal. *Sci. Rep.* **6**, 24716; doi: 10.1038/srep24716 (2016).

## Supplementary Material

Supplementary Information
