# Supplementary material for: Molecular Structures of Al/Si and Fe/Si Coprecipitates and the Implication for Selenite Removal
Source: Sci Rep. 2016 Apr 20;6:24716. doi: 10.1038/srep24716 (PMC4837376; doi:10.1038/srep24716)
Supplement: Supplementary Information [file srep24716-s1.pdf]

Supporting Information

Molecular Structures of Al/Si and Fe/Si Coprecipitates and the Implication for Selenite Removal

Ya-Ting Chan[1], Wen-Hui Kuan[2], Yu-Min Tzou[1], Tsan-Yao Chen[3], Yu-Ting Liu[1]*, Ming-Kuang Wang[4], Heng-Yi Teah[5]

[1]Department of Soil and Environmental Sciences, National Chung Hsing University, 250 Kuo Kuang Rd., Taichung 40227, Taiwan, R.O.C.
[2] Department of Safety, Health and Environmental Engineering, Ming-Chi University of Technology, New Taipei City 24301, Taiwan, R.O.C.
[3] Department of Engineering and System Sciences, National Tsing Hua University, Hsin-Chu 30043, Taiwan, R.O.C.
[4] Department of Agricultural Chemistry, National Taiwan University, Taipei 10617, Taiwan, R.O.C.
[5] Division of Environmental Studies, Graduate School of Frontier Sciences, The University of Tokyo, 332 Building of Environmental Studies, 5-1-5 Kashiwanoha, Kashiwa City, Chiba 277-8563, Japan

[*]Corresponding authors:
Department of Soil and Environmental Sciences, National Chung-Hsing University, 250 Kuo Kuang Rd., Taichung 40227, Taiwan, R.O.C.
Tel.: +886-4-2284-0373 ext. 3402; fax: +886-4-22855167; Email: yliu@nchu.edu.tw

Number of Supporting Information (SI) Pages – 5 (including cover sheet)
Number of SI Figure – 2
Number of SI Table – 2

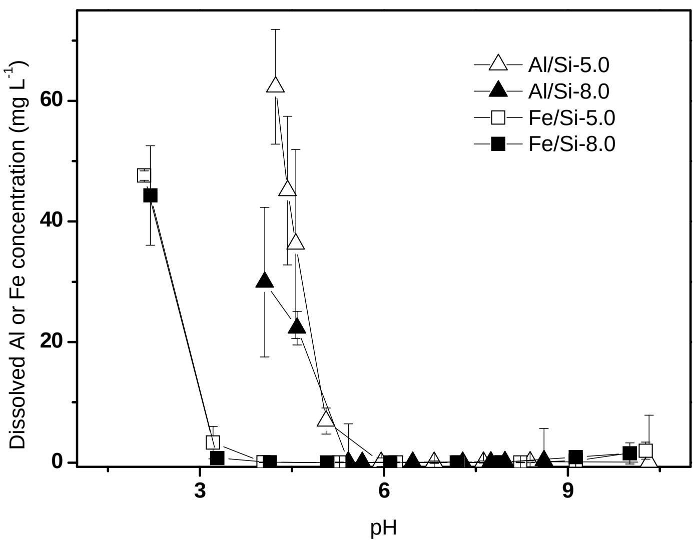

Figure S1. Trends of Al or Fe dissolution from Al/Si and Fe/Si coprecipitates synthesized at pH 5.0 (Al/Si-5.0, Fe/Si-5.0) and 8.0 (Al/Si-8.0, Fe/Si-8.0) under an electrolyte concentration of 0.01 M $NaNO_3$.

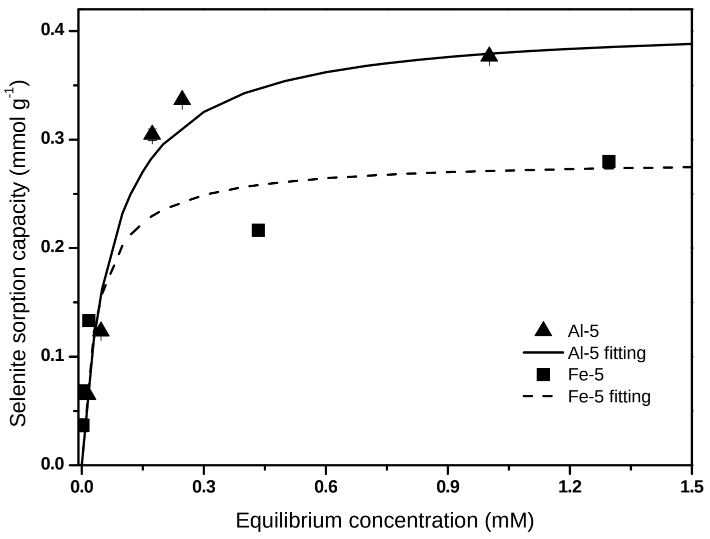

Figure S2. Results of isotherm sorption of selenite on Al and Fe precipitates synthesized at pH 5.0 (Al-5.0 and Fe-5.0) at 25 °C under a 0.01 M $NaNO_3$ background. The Langmuir fitting results showed the maximum adsorption capacities for Al and Fe precipitates were 0.41 and 0.28 mmol g^{-1}. The coefficients of determination for the fitting are 0.996 and 0.992 ($P < 0.05$, n=5), respectively.

Table S1. The BET surface areas for SiO_2, Al-, Fe-hydroxides and Al/Si, Fe/Si coprecipitates synthesized at pH 5.0 and 8.0 (Al or Fe/Si-5.0 and -8.0) under an electrolyte concentration of 0.01 M $NaNO_3$.

System	S_{BET}[a] (m^2 g^{-1})
SiO_2	196
Pure hydroxides	
Al-5.0	54
Al-8.0	158
Fe-5.0	283
Fe-8.0	284
Coprecipitates	
Al/Si-5.0	155
Al/Si-8.0	188
Fe/Si-5.0	256
Fe/Si-8.0	233

[a] Specific surface area determined by the BET method.

Table S2. Elemental compositions in atomic percentage for SiO_2 and Al/Si, Fe/Si coprecipitates synthesized at pH 5.0, 6.5, and 8.0 (Al/Si or Fe/Si-5.0, -6.5, -8.0) under an electrolyte concentration of 0.01 M $NaNO_3$.

Sample	Al2p (%)	Si2p (%)	Al/Si ratio	Sample	Fe2p (%)	Si2p (%)	Fe/Si ratio
SiO_2	-	28.8	-		-	-	-
Al/Si coprecipitates				Fe/Si coprecipitates			
Al/Si-5.0	3.9	22.9	0.17	Fe/Si-5.0	1.6	25.5	0.06
Al/Si-6.5	6.0	21.6	0.28	Fe/Si-6.5	1.3	18.2	0.07
Al/Si-8.0	7.2	21.5	0.33	Fe/Si-8.0	1.8	25.4	0.07
